# The Effects of Selective Muscle Weakness on Muscle Coordination in the Human Arm

**DOI:** 10.1155/2018/5637568

**Published:** 2018-09-19

**Authors:** Jinsook Roh, Randall F. Beer, Andrew Lai, Monica Rho, Kristopher R. Karvelas, Antoun M. Nader, Mark C. Kendall, William Z. Rymer

**Affiliations:** ^1^Department of Biomedical Engineering, University of Houston, Houston, TX 77004, USA; ^2^Department of Kinesiology and Neuromotor Science Program, Temple University, Philadelphia, PA 19122, USA; ^3^Department of Physical Medicine and Rehabilitation, Feinberg School of Medicine, Northwestern University, Chicago, IL 60611, USA; ^4^Department of Biomedical Engineering, Northwestern University, Chicago, IL 60611, USA; ^5^Sensory Motor Performance Program, Rehabilitation Institute of Chicago, Chicago, IL 60611, USA; ^6^Department of Physical Medicine and Rehabilitation, Wake Forest Baptist Medical Center, Winston-Salem, NC 27157, USA; ^7^Department of Anesthesiology, Feinberg School of Medicine, Northwestern University, Chicago, IL 60611, USA; ^8^Department of Anesthesiology, Rhode Island Hospital, Warren Alpert Medical School of Brown University, Providence, RI 02903, USA

## Abstract

Despite the fundamental importance of muscle coordination in daily life, it is currently unclear how muscle coordination adapts when the musculoskeletal system is perturbed. In this study, we quantified the impact of selective muscle weakness on several metrics of muscle coordination. Seven healthy subjects performed 2D and 3D isometric force target matches, while electromyographic (EMG) signals were recorded from 13 elbow and shoulder muscles. Subsequently, muscle weakness was induced by a motor point block of brachialis muscle. Postblock subjects repeated the force generation tasks. We quantified muscle coordination pre- and postblock using three metrics: tuning curve preferred direction, tuning curve area, and motor modules analysis via nonnegative matrix factorization. For most muscles, the tuning direction for the 2D protocol was not substantially altered postblock, while tuning areas changed more drastically. Typically, five motor modules were identified from the 3D task, and four motor modules were identified in the 2D task; this result held across both pre- and postblock conditions. The composition of one or two motor modules, ones that involved mainly the activation of shoulder muscles, was altered postblock. Our results demonstrate that selective muscle weakness can induce nonintuitive alternations in muscle coordination in the mechanically redundant human arm.

## 1. Introduction

Selective muscle weakness is a common effect of various musculoskeletal injuries, neurological injuries, and surgical procedures. As examples, focal motor mononeuropathy, muscle/tendon tears and ruptures, plexopathy, and radiculopathy are all conditions that can cause selective muscle weakness. Similarly, tenotomy, that is, surgical transection of a tendon, can lead to weakness of associated muscles [1]. However, it is less clear whether selective muscle weakness also impacts intermuscular coordination. This is a critical issue, because skilled motor performance typically requires the coordinated activation of multiple muscles, and this coordination could be disrupted in pathological ways. Clinically, understanding the potential changes in muscle coordination associated with focal muscle weakness may be extremely helpful. Physicians and therapists could give patients reasonable expectations of neuromuscular compensation and target their rehabilitation protocols towards improving strength and coordination of the muscles that facilitate maximum functional recovery.

The few previous studies of selective muscle weakness have generally concluded that it does not impact muscle coordination. One such study used electrical stimulation to induce muscle fatigue, demonstrating that the electromyographic (EMG) spatial tuning of wrist muscles is robust to selective muscle impairment, both simulated and real [2]. In the study, the tuning direction of wrist muscles was not altered following electrical stimulation, which led to the conclusion that muscle coordination is habitual rather than optimal. However, the wrist joint is actuated by five muscles that have minimal overlap in function, resulting in limited mechanical redundancy and hence limited potential for coordination to adapt [3]. Thus, a more comprehensive study of the reorganization of muscle activity in a more redundant musculature following selective muscle weakness is warranted.

In the recent literature, dimensionality reduction techniques have been widely used to quantify muscle coordination and to detect changes in muscle coordination following a natural or experimental event or procedure, including in pathological conditions (e.g., stroke [4–16]), cerebral palsy [17–19], Parkinson's disease [20, 21], spinal cord injury [22–24], pain [25–27], or practicing sports [28–33]). These techniques represent complicated, high-dimensional muscle activations using a small number of motor modules, effectively reducing muscle activation dimensionality. As defined here, these motor modules are “muscle synergies,” characteristic patterns of muscle activity that can be flexibly combined to produce functional motor behaviors. Of particular relevance to the present study, dimensionality reduction techniques have been extensively tested and applied to human reaching [4, 5, 15, 26, 34–38] and isometric force generation in the upper extremity [6, 7, 39–41].

In this study, we quantified shoulder and elbow muscle coordination underlying isometric force tasks performed pre- and postinduction of selective weakness of the brachialis muscle. We aimed to examine the effects of selective motor point block in the case of a pure elbow flexor (monoarticular muscle), with the greatest contribution (about 50%; [42]) to the elbow flexion torque, so that the block would potentially maximize the change in the capability of isometric flexion force generation in the human arm. Dimensionality reduction techniques and other metrics were used to provide succinct representations of EMG patterns. In the past decade, the use of ultrasound guidance for peripheral nerve blockade has become increasingly popular in medical practice [43]. The visualization of neurovascular structures and surrounding anatomy has allowed more precise and accurate needle placement techniques [44, 45]. Accordingly, we induced selective muscle weakness via lidocaine injection to avoid potentially confounding effects associated with electrical stimulation (e.g., changes in reflex and central nervous system function and metabolic/thermal effects [46, 47]). We hypothesized that muscle coordination during isometric force generation would be altered following selective muscle weakness; this hypothesis was supported by the discovery of altered tuning curve areas and altered motor modules postblock.

## 2. Methods

### 2.1. Participants

Seven healthy, right-handed volunteers (age, 24.1 ± 4.3 years; 2 females) provided written consent prior to participation in the study. Exclusion criteria were as follows: (1) a history of orthopaedic injuries, neuromuscular disorders, or musculoskeletal pain affecting the upper extremity; (2) a history of an allergic reaction to the local anesthetic used for a dental or medical procedure; (3) cardiovascular or hepatic disease; and (4) women who were pregnant or nursing. All experimental procedures were approved by the Northwestern University Institutional Review Board, Chicago, IL (IRB number STU 00064439) and performed in accordance with the Declaration of Helsinki.

### 2.2. Equipment

**Figure 1:**
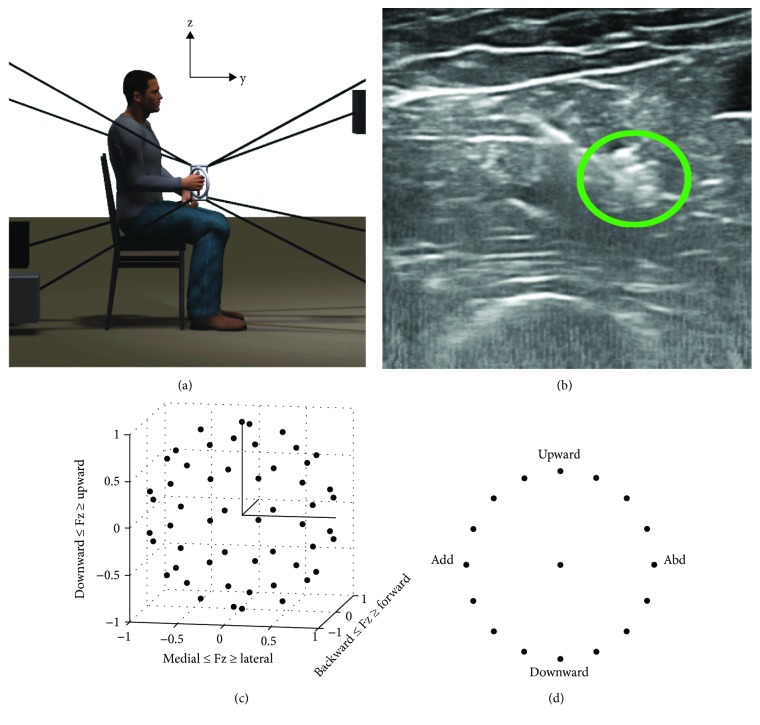
Experimental setup and design. (a) Lateral view of the experimental setup. A cable robot (shown schematically) was used to record the hand position and 3D forces generated at the hand (see [48, 49] for details concerning the robot). A subject grasps the central end-effector (a gimbaled handle mounted on a six degrees of freedom load cell) via cables (depicted by black lines) connected to a spatial array of motors (indicated by black filled squares). The right-handed coordinate system (i.e., X-axis is out of the page) is indicated at the top center. (b) Ultrasonographic view of the brachialis muscle. The green circle indicates the position of the needle tip, inserted to inject lidocaine. (c) The distribution of 54 targets for the 3D isometric force target matching protocol. Force targets (black filled circles) were homogeneously distributed to avoid bias in force direction. (d) The distribution of 16 targets for the 2D force target matching protocol. Force targets (filled black circles) were defined on the circumference of a circle in the frontal plane. The direction information is indicated for the right arm.

The Multi-Axis Cartesian-based Arm Rehabilitation Machine (MACARM), a cable (wire) robot for upper limb research and rehabilitation, was used to measure three-dimensional (3D) forces and to position the hand during isometric force target matching. The MACARM (Figure 1(a)) was comprised of eight active modules anchored at the corners of a cubic frame. The end-effector for this robot incorporated a gimbaled handle mounted on a six degrees of freedom (DOF) load cell (JR3 model number 45E15A). Additionally, the MACARM supported the collection of limb orientation data using a three DOF orientation sensor (Xsens Technologies BV, The Netherlands). This sensor was strapped to the upper arm to measure shoulder rotation. Forces and position/orientation data were sampled at 64 Hz and stored on a computer for subsequent analyses.

### 2.3. Data Collection

Both surface and intramuscular electromyographic (EMG) signals were recorded (Delsys Incorporated, Boston, MA, USA) at 1920 Hz from up to 13 muscles acting at the elbow and/or shoulder. These muscles included brachioradialis; pronator teres; brachialis; biceps brachii and short and long heads; supinator; triceps brachii and lateral and long heads; anconeus; anterior, medial, and posterior deltoid; and pectoralis clavicular fibers. For subject comfort, surface EMG electrodes were used to record EMGs unless cross-talk from adjacent muscles was a concern. Intramuscular EMG recordings were used for anconeus, supinator, brachialis, and pronator teres. Intramuscular EMGs were recorded by using bipolar electrodes made of 50-micron diameter nylon-coated nickel/chromium wires inserted into the muscle belly using sterile 25-gauge hypodermic needles. Electrode placement followed standard guidelines [50, 51].

To examine whether maximum end-point forces were altered following motor point block and to confirm that the block effect lasted until the end of data collection, subjects generated maximum voluntary contractions (MVC) in the ±X-, ±Y-, and ±Z-directions (see Figure 1(c)) at three different stages of the experiment: prior to (pre-MVC), 10 minutes after the injection of lidocaine into a targeted muscle (post-MVC1) as well as at the end of data collection (post-MVC2). For each stage, three repetitions were performed in each direction. We adopted a task-dependent, rather than muscle-specific, measure of the MVC since EMG normalization (unit variance normalization) for identification of motor modules did not depend on the max EMG value and the six-directional task-dependent measure reduced measurement time and the potential for muscular fatigue, as compared to 13 muscle-specific measures. In addition, considering the known mechanical action of brachialis, the MVC measurement was expected to well approximate the maximal EMG of the targeted muscle. The measurement of maximum forces allowed subject-specific scaling of target force magnitude and was used to calculate the decrease in the magnitude of the output force following lidocaine injection. Based on Roh et al. [39], the lateral force direction, that is, +X for the right arm, was expected to be the weakest direction for all subjects. Accordingly, the magnitude of force targets for each subject was set as 40% of maximum lateral force (MLF).

### 2.4. Experimental Design

#### 2.4.1. Motor Point Block

Ultrasound-guided motor point blockade of the brachialis, a major elbow flexor, was performed by a board-certified anesthesiologist. The approximate motor point locations of the target muscle were initially identified relative to anatomical landmarks [52–57]. To account for the substantial intersubject variability in the number and location(s) of motor points for the target muscle, ultrasound imaging was then used to identify the motor points for each subject and assist in needle positioning (Figure 1(b)). Subsequently, a sterile needle was directed at each motor point and 2% lidocaine injected (total injected volume: 4.21 ± 2.48 ml; *n* = 7, mean ± STD) during visualization by ultrasonography. The anesthesiologist monitored cardiovascular and respiratory vital signs and the participant's state of consciousness for the first 30 minutes after the injection. Successful motor point block was confirmed qualitatively by observing the decrease of upward directional force and EMG amplitude of the targeted muscle and quantified using two metrics (see Data Analysis).

#### 2.4.2. Force Target Matching Protocol

Subjects performed two force target matching protocols (3D and 2D) with their dominant (right) arm under isometric conditions, pre- and postlidocaine injection. During the 3D protocol, subjects were expected to match 54 force targets approximately uniformly distributed in 3D force space to maximize the variability of EMG patterns as well as avoid any bias in target force direction (Figure 1(c)). The 2D protocol required subjects to match 16 force targets equally spaced in the frontal plane (Figure 1(d)) and was repeated six times. The results of the 2D protocol were used to examine how each muscle's activation was tuned in force space. During target matching for both protocols, subjects were seated in an adjustable salon chair and grasped the MACARM's gimbaled handle, which was positioned so that the arm was in a parasagittal plane aligned with the shoulder with the upper arm segment oriented vertically and the elbow flexed to 90 degrees. Wrist and trunk movements were restrained using a commercially available brace and strapping, respectively. Changes in shoulder position were monitored by using a laser pointer directed at the acromion and verbally corrected if necessary. EMG and three-dimensional forces were recorded for further analyses.

For each trial, the target force was indicated on a computer monitor, with real-time feedback of the force generated at the hand provided by a spherical cursor. A successful target match required the subject to maintain the center of the cursor in the target zone (a sphere around the target force with a radius equal to 20% of the targeted force magnitude) for one second. Three attempts at a match were allowed before proceeding to the next target in a random sequence. An intertrial interval of 10 s and a one-minute rest after each block of 10 trials were provided to minimize the potential for fatigue.

### 2.5. Data Analysis

#### 2.5.1. EMG Preprocessing: MVC

To identify the maximum EMG magnitude of each muscle during MVC trials, we demeaned, rectified, and averaged the EMG signals using a 500 ms sliding window. From the processed EMG, the maximum EMG amplitude was selected, and the mean baseline EMG was subtracted (baseline EMG was recorded while subjects grasped the handle without force generation) to calculate the maximum EMG amplitude recorded during the MVC performance.

#### 2.5.2. EMG Preprocessing: Target Matching

We demeaned, rectified, and then averaged EMG signals over the one-second target matching phase. To examine the task-relevant EMG signals, the mean baseline EMGs (recorded while the subject grasped the handle without force generation) were removed from the averaged data. When negative values occasionally resulted after subtraction of the baseline EMGs, the values were set to zero to meet the positivity constraint of motor module identification using nonnegative matrix factorization (NMF) [58, 59]. Resultant EMG data for each trial were a vector whose dimension was the number of muscles recorded, and these data reflected the increase in muscle activity corresponding to active force production. Prior to module extraction, the EMG data recorded from each muscle were concatenated across trials relevant to the purpose of the module extraction.

#### 2.5.3. Quantifying Efficacy of Motor Point Block

The efficacy of the neuromuscular block, pharmacologically induced by injection of lidocaine, was assessed by the changes in the amplitude of the +z force (which required the generation of elbow flexion torque) and the amplitude of BRA EMG recorded during MVC trials. In addition, we measured the changes in BRA EMG recorded during 2D force matches. The reason why we quantified the effects of lidocaine injection in that way was because we intended to measure both the decrease in the capacity of maximal force generation in the mechanically corresponding direction and the degree to which the EMG amplitude would decrease in an actual motor task.

The maximal value of the upward directional force and BRA EMG measured during MVC trials preblock was compared to those of postblock, respectively, to quantify the effect of the block on the elbow flexors. For each subject, the maximal force and BRA EMG amplitudes were averaged across the MVC trials collected at the three different stages (pre-MVC, post-MVC, and post-MVC2; see Data Collection). The ratio of post-MVC or post-MVC2 to pre-MVC was calculated for a comparison across three conditions. In addition, for the 2D protocol, we identified the force direction with the largest average preblock BRA activation and then computed the ratio of postblock to preblock BRA EMG for that target.

#### 2.5.4. Constructing Tuning Curves and Analyzing Preferred Directions and Tuning Areas

For the 2D protocol, we generated muscle tuning curves and polar plots of muscle activations for different force directions, to characterize muscular coordination qualitatively [60, 61]. Tuning curves were constructed separately for the pre- and postblock conditions. Following preprocessing, muscle activations were plotted as a function of the 16 target force directions.

For quantitative analysis, we determined the preferred direction and area for each muscle's tuning curve, pre- and postblock. To calculate preferred direction, each point on the tuning curve was treated as a vector. The preferred direction was defined as the vector sum across all 16 points in the tuning curve [61]. The angle between the positive *x*-axis and the resulting vector was defined as the preferred angle of the tuning curve. Tuning curve area was defined as the area inside the tuning curve shape. To calculate tuning curve area, each point on the tuning curve was considered as a vertex of a polygon. The polygon area was computed using MATLAB.

#### 2.5.5. Identifying Motor Modules

For each trial, EMGs for each muscle were averaged over the period of stable force generation (i.e., the last second of each successful target match trial) and represented as a single vector whose dimension was the number of muscles examined. For each muscle, the pooled EMGs across trials were normalized to have unit variance prior to the identification of motor modules, which ensured that subsequent motor module identification from preprocessed EMGs was not biased towards high-variance muscles [7]. After normalization by the same factor, the standard deviation of the combined pre- and postblock EMG data and the pooled EMG data were separated into pre- and postblock EMG data, respectively, to identify motor modules from each dataset. BRA was excluded from this analysis, because including BRA would introduce distortion of the motor modules given that each muscle activation was normalized to have unit variance across trials to prevent a bias of identified modules toward muscles with a large variance.

We modeled EMG patterns (EMG_isometric_) as linear combinations of a set of *N* motor modules (*W*_isometric_), each of which specified the balance of activation across recorded muscles [4, 6, 62–68]:

(1) $$\begin{array}{c} {EMG}_{isometric}=W_{isometric}\cdot C_{isometric}, \end{array}$$

where *W*_isometric_ is an *M* (number of muscles examined) by *N* matrix, containing the *N* motor modules (of unit magnitude) in each column, and *C*_isometric_ is an *N* by *T* (number of trials) matrix, with each column containing the activation coefficients of each module for a specific trial. A nonnegative matrix factorization (NMF) algorithm [58, 59] was applied to either the pre- or postblock EMG dataset for each subject to identify the minimum number of motor modules which captured most of the total data variance.

#### 2.5.6. Estimating the Number of Motor Modules

To identify the minimum number of motor modules that adequately predicted the spatial characteristics of a given EMG dataset, we first calculated variance accounted for (VAF) based on the entire dataset (global VAF). Here, the total data variation, defined as the trace of the covariance of the EMG data matrix, was used to define a multivariate VAF measure:

(2) $$\begin{array}{c} VAF=100\;\times \;\left(1-\left(\frac{SSE}{SST}\right)\right), \end{array}$$

where SSE is the sum of the squared residuals, and SST is the sum of the squared EMG data (uncentered data; see [69]). The identification of motor modules was repeated 100 times to characterize the distribution of global VAF values. The number of modules underlying each dataset was defined as the minimum number of motor modules required to achieve a mean global VAF > 90%, while satisfying local criteria of fit (see below), subject to the requirement that adding another motor module increased the mean global VAF less than 5%. As a local criteria, we required the mean VAF for each muscle (muscle VAF) to exceed 75%. Muscle VAF was computed in 2, based on the EMG signals of individual muscles. This procedure ensured that the estimated number of modules adequately reconstructed each muscle's activation, as well as the overall data.

#### 2.5.7. Quantifying Similarity of Motor Modules

The similarity between the motor modules underlying two datasets (e.g., pre- and postblock motor modules identified from 3D isometric force generation) was calculated by using the following metrics: *r* values, that is, scalar product of two motor module vectors, shared subspace dimensionality [25, 26, 64, 68] and global reconstruction VAF values [6, 7, 64, 70]. While the *r* value was based on direct comparison of individual motor modules, the other metrics were rather holistic measures of similarity because they considered the set of motor modules as a whole. To calculate the *r* values between motor module vectors (i.e., unit vectors) from two module sets, modules were matched across sets to maximize the scalar product between them. In addition, the similarity between the sets of modules underlying two datasets was computed by calculating the global VAFs obtained by cross-fitting the motor modules, that is, using the modules for dataset A to reconstruct dataset B and vice versa.

#### 2.5.8. Statistical Analysis

We tested the effectiveness of the motor point block by using a one-sample *t*-test to examine whether the mean of the ratio of post-MVC or post-MVC2 to pre-MVC was different from one. Prior to the test, data normality was verified by using the Anderson-Darling test with data pooled across all subjects. Similarly, we examined whether the pre- and postblock EMGs of BRA differed by pooling the BRA data across all seven subjects, verifying normality using the Anderson-Darling test and applying a one-sample *t*-test to test whether the ratio of EMG activity was different from 1.

To evaluate the statistical significance of the global and muscle VAF measures, random modules were generated by randomly sampling the EMG amplitudes independently for each muscle, from the empirical distribution of the EMG dataset [35]. The random modules were normalized to be a unit vector and used to fit the original EMG data to determine the quality of reconstruction by chance (random VAF value). This procedure was repeated 200 times for each EMG dataset to define the distribution of random VAF values.

As an attempt to test whether the composition of motor modules was consistent across the repeated trials for the 2D protocol, we randomly assigned three of the six repetitions performed for each target to each of two datasets. Motor modules were identified from the two subdatasets, respectively. Cross-reconstruction VAF values were calculated to compare whether the VAF values were different between the two subdatasets for both the pre- and postblock conditions within the same subject.

## 3. Results

### 3.1. Block Efficacy

**Figure 2:**
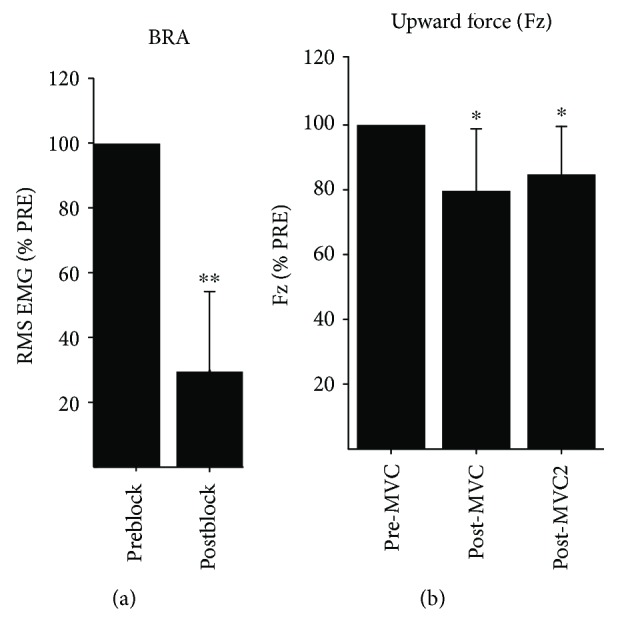
Injection of lidocaine decreased the EMG magnitude of brachialis (^∗∗^*p* < 0.01) and end-point force in the upward direction (^∗^*p* < 0.05). The effect of lidocaine injection last to the end of the experiment (^∗^*p* < 0.05). (a) The ratio of postblock EMG to preblock EMG in brachialis (BRA) based on results for the 2D protocol. (b) The ratio of upward directional forces (Fz), measured at three different stages of the experiment, to the preblock force. The three stages of recording included prior to (pre-MVC) and after lidocaine injection (post-MVC) and after finishing postblock data collection (post-MVC2). All forces were measured during maximal voluntary contraction (MVC) trials.

The injection of lidocaine resulted in decreased BRA muscle activation amplitude and vertical end-point force across the seven subjects. For example, the 2D protocol lidocaine injection reduced BRA EMG by 70.7 ± 25.4% (mean ± SD; *n* = 7; ^∗∗^*p* < 0.01) (Figure 2(a)). In addition, the upward directional force (Fz) during MVC trials decreased by 20.4 ± 19.2% (mean ± SD; *n* = 7; ^∗^*p* < 0.05) (Figure 2(b)). The decrease in Fz lasted to the end of the experiment, at which point the force amplitude was decreased by 15.3 ± 14.8% (mean ± SD; *n* = 7; ^∗^*p* < 0.05), as compared to the preblock condition. Similarly, the EMG amplitude of BRA measured during MVC trials decreased by 45% in the postblock condition, ranging from 26% to 64%.

### 3.2. The Effects of Muscle Block on Tuning Curves, Tuning Directions, and Tuning Areas of Individual Muscles

**Figure 3:**
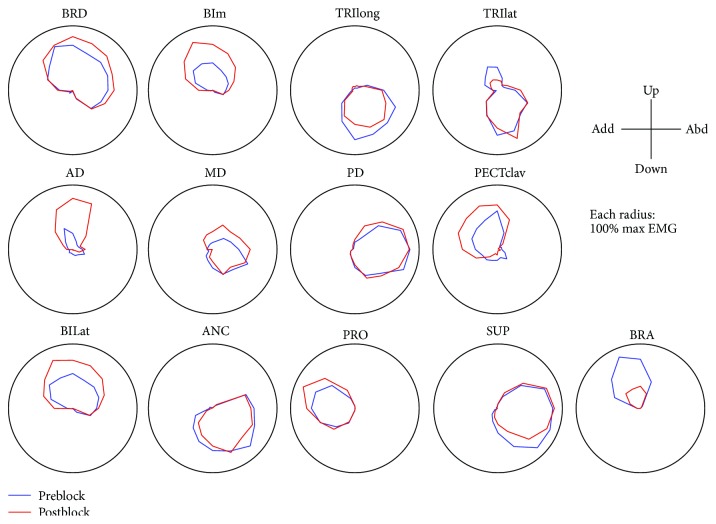
Average tuning curves and muscle activation pattern as a function of target force direction, for the 2D protocol for a single subject in pre- and postblock (blue and red, resp.). The tuning areas, but not many tuning directions, were altered following BRA motor point block. The coordinate system for force directions is indicated at the top right corner. The EMG of each muscle was normalized by the maximum EMG of the muscle in the entire dataset recorded from the subject. Muscle names are indicated in an abbreviated form (BRD: brachioradialis; BIm: short head of biceps brachii; TRIlong and TRIlat: long and lateral heads of triceps brachii, resp. AD, MD, and PD: anterior, medial, and posterial fibers of deltoid, resp. PECTclav: clavicular fibers of pectoralis major: BILat: long head of biceps brachii; ANC: anconeus; PRO: pronator teres; SUP: supinator; BRA: brachialis).

Representative pre- and postblock tuning curves are shown in Figure 3. Preferred directions and tuning areas of individual muscles are summarized across subjects in Table 1. Generally, only minor changes in preferred direction were found for most muscles following motor point block of BRA. Statistically significant changes in the preferred direction of one or more muscles were found in five out of seven subjects (Table 1, *p* < 0.05). However, though the change of preferred direction in certain muscles was statistically significant, the magnitude of the change was typically small; across 13 muscles of the seven subjects in the group, only three out of 91 tuning curves showed significant changes (*p* < 0.05) in tuning direction of 10 degrees or more. The muscle groups with minor changes in the preferred direction postblock were subject-specific; alterations were identified in the shoulder as well as elbow muscles.

All seven subjects showed significant changes in the tuning areas of at least two muscles following BRA motor point block (Table 1). Following the block, the tuning area of BRA activation decreased by over 48% up to 100%, as compared to the preblock condition. The tuning area of one or more synergistic muscles of BRA, such as BRD, BIm, and BIlat, tended to scale up to compensate for the partial loss of BRA activation in six out of seven subjects, as illustrated in Figure 3. The magnitude of the change in the tuning area was subject-specific. Surprisingly, the tuning area of one or more elbow extensors (i.e., TRIlong, TRIlat, and ANC), antagonistic muscles of the weakened one, significantly decreased in five out of seven subjects. The tuning area of SUP whose mechanical action was matched to that of BI decreased in four out of five cases collected in the experiment, though the magnitude was not statistically significant (*p* > 0.05). The tuning area of PRO whose mechanical action was opposite to BI increased in six out of seven cases with one exceptional increase, potentially due to change in the intramuscular EMG electrode location within the muscle. Additionally, several subjects exhibited statistically significant changes in the tuning areas of shoulder muscles, though the direction of change was not consistent.

### 3.3. Dimensionality of 3D and 2D Force Generation Tasks

**Figure 4:**
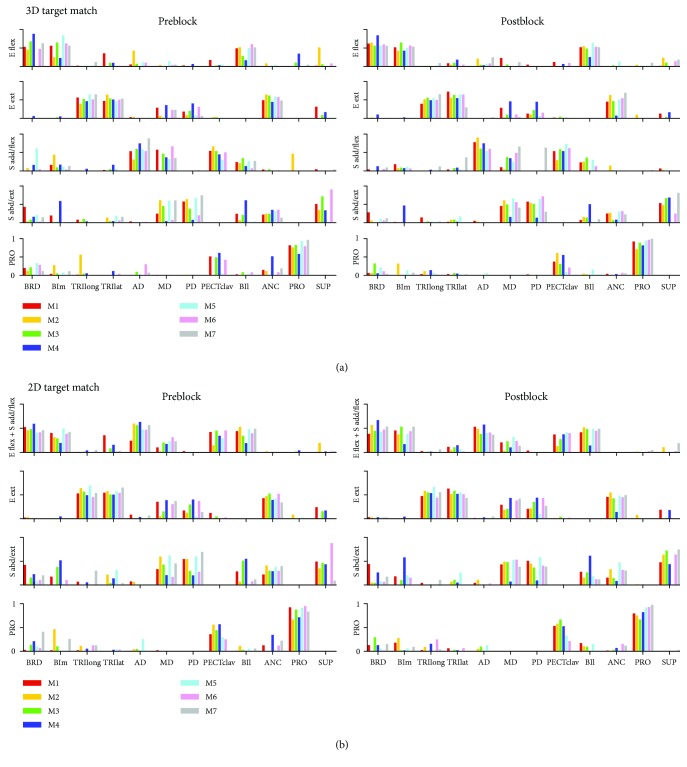
Structure of motor modules for individual pre- and postblock conditions in 3D and 2D force target matches (*n* = 7 subjects; M1–M7). Each subject's modules are differently color-coded. (a) Five motor modules identified from EMGs recorded during 3D force target matches pre- and postblock. (b) Four motor modules identified from EMGs recorded during 2D force target matches pre- and postblock. The title of motor modules was indicated in an abbreviated form (E flex: elbow flexor; E ext: elbow extensor; S add/flex: shoulder adductor/flexor; S abd/ext: shoulder abductor/extensor; PRO: pronator-dominant one. E flex + S add/flex pattern is the one as if the two module vectors. E flex and S add/flex are mathematically summed together. Muscle names are indicated in an abbreviated form (BRD: brachioradialis; BIm: short head of biceps brachii; TRIlong and TRIlat: long and lateral heads of triceps brachii. AD, MD, and PD: anterior, medial, and posterial fibers of deltoid. PECTclav: clavicular fibers of pectoralis major; BILat: long head of biceps brachii; ANC: anconeus; PRO: pronator teres; SUP: supinator; BRA: brachialis).

Typically, five and four motor modules were identified from the muscle activation patterns recorded pre- and postblock for the 3D and 2D force matching protocols, respectively (Figure 4). While the 3D protocol required 5.00 ± 0.93 and 4.86 ± 0.64 modules in the pre- and postblock conditions, the 2D protocol required 4.14 ± 0.35 and 4.29 ± 0.45 modules pre- and postblock, respectively (*n* = 7; mean ± STD). The number of motor modules was not significantly different between the pre- and postblock conditions for either protocol (ANOVA, *F*_(1,12)_ = 0.14, *p* > 0.05 and *F*_(1,12)_ = 0, *p* > 0.05, for the 3D and 2D protocols, resp.). The global VAF values were 94.0 ± 1.7% with five modules for the 3D protocol and 94.7 ± 1.3% with four modules for the 2D protocol (*n* = 7). All VAF values were statistically greater than the chance level (*p* < 0.05). Five and four modules could account for most of the EMG variance across all subjects for the 3D and 2D protocols, respectively. Accordingly, to facilitate comparisons within and across subjects, we identified five and four modules from each subject's 3D and 2D EMG data.

The composition of the five motor modules identified from 3D force matches under isometric conditions involved the activation of a distinctive group of muscles in both pre- and postblock conditions (Figure 4(a)). Two modules were dominated by the activation of elbow flexors (BRD, BIm, and BIl) and elbow extensors (TRIlong, TRIlat, and ANC), respectively. The third one, “shoulder adductor/flexor (S add/flex)” module, involved the activation of AD, MD, PECTclav, and BIl. The shoulder abductor/extensor pattern included the activation of MD, PD, SUP, and some elbow muscles. The remaining pattern was dominated by the activation of PRO and that of PECTclav to a lesser degree.

Three of four typical motor modules (E ext, S abd/ext, and PRO), identified from 2D force matches in the subject group, were similar to those identified from 3D force matches (Figure 4(b)) in both the pre- and postblock conditions. The remaining one, “E flex + S add/flex” module, included the activation of BRD, BIm, AD, MD, PECTclav, and BIl. A separate analysis showed that this module appeared to be a linear combination of the E flex and S add/flex modules identified for the 3D protocol. To identify the degree to which the motor modules were conserved following the pharmacologically induced muscle weakness, we examined the similarity of motor modules quantitatively.

### 3.4. Similarity of Pre- and Postblock Motor Modules Conditions

**Figure 5:**
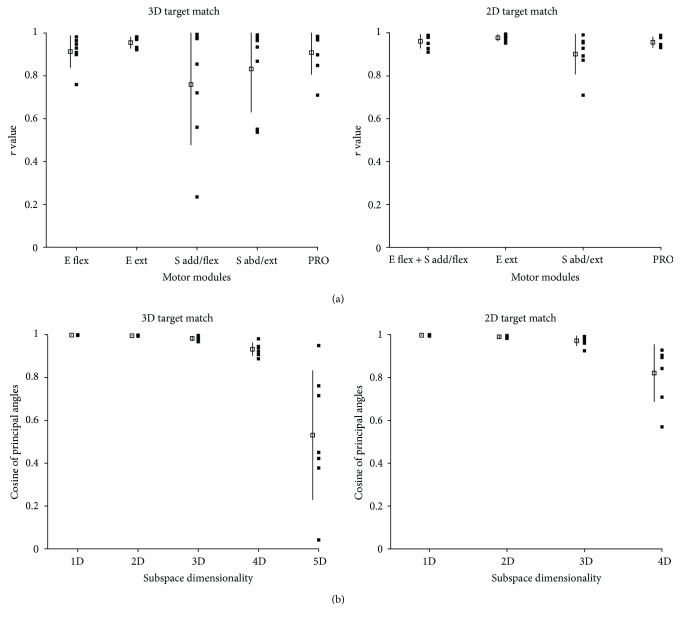
Indices of similarity of motor modules between pre- and postblock conditions in 3D and 2D force target matches in the group of selective muscle weakness. For each module, the similarity indices were indicated as mean and SD (open shape and bar; *n* = 7) as well as the distribution (filled shapes) of individual subject's data. Note that injection of lidocaine-induced alterations in certain motor modules when the injection weakened the activity BRA. (a) The results of scalar product between pre- and postblock modules (*r* values). (b) Cosine of the principal angles (CPA) of subspace spanned by pre- and postblock modules. If pre- and postblock modules were equal, the cosine of the principal angles would be one for all dimensions.

The metrics used to quantify the similarity of pre- and postblock motor modules revealed that selective BRA weakness could induce alterations in certain motor modules.  Figure 5(a) shows the *r* values, that is, the results of scalar products of pre- and postblock motor modules within the same subject (*n* = 7), for 3D and 2D force target matches, respectively. Interestingly, the BRA block mainly induced alterations in the modules involving activation of proximal muscles such as S add/flex and S abd/ext modules underlying 3D target matches. The mean *r* values for these modules were 0.76 and 0.83, respectively, while the *r* values for other modules exceeded the similarity threshold (0.9). Likewise, the results of subspace dimensionality analysis showed that the cosine of at least one of the five principal angles (CPA) was less than the threshold (0.9) for six of the seven subjects (Figure 5(b)). These results consistently indicate that one to three out of five motor modules were altered following selective muscle block for 3D force generation. In the case of 2D protocol, the four *r* values, on average, were >0.9 (*n* = 7 subjects). In addition, the CPA of the first three and four angles out of four angles was >0.9 for *n* = 4 and 3 subjects, respectively. This result shows that minimal alterations of motor modules were observed postblock for 2D force target matches.

**Figure 6:**
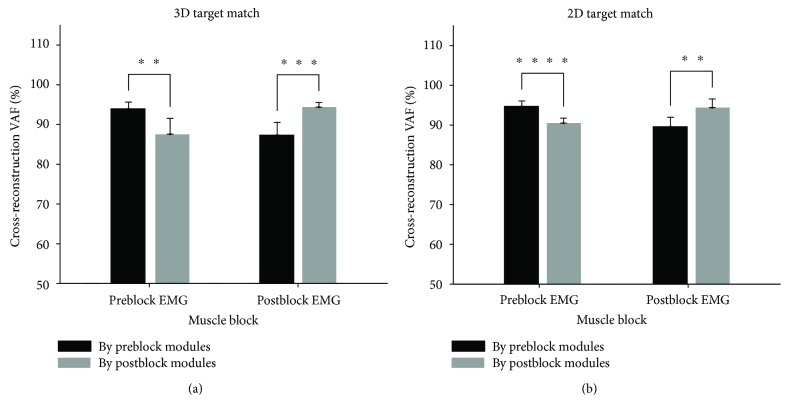
Cross-reconstruction VAF values as an index of similarity between pre- and postblock motor modules in 3D and 2D force target matches. If preblock and postblock motor modules were identical, the cross-reconstruction VAF values of the two conditions (when one EMG dataset was reconstructed by preblock modules (black) and when the same dataset was reconstructed by postblock modules (gray)) would be the same. Note that the structure of motor modules was altered following lidocaine injection in either 3D or 2D force target matches (*n* = 7). (a) Cross-validation VAF values in 3D force target matches. (b) Cross-validation VAF values in 2D force target matches. (^∗∗^*p* < 0.01; ^∗∗∗^*p* < 0.001; ^∗∗∗∗^*p* < 0.0001).

Comparisons of motor modules as a group also support alterations of motor modules following lidocaine injection.  Figure 6 shows that the cross-reconstruction VAF values were significantly smaller when modules of the preblock condition were used to reconstruct the EMG of the postblock case (second black bar in Figure 6(a)), as compared to the case of postblock EMG reconstruction by the postblock modules (second gray bar in Figure 6(a); ^∗∗∗^*p* < 0.001) during 3D force target matches. Similarly, for the 2D protocol cross-reconstruction, VAFs were significantly different from the reconstruction VAFs (^∗∗^*p* < 0.01; ^∗∗∗∗^*p* < 0.0001), indicating that motor modules were altered (Figure 6(b)).

## 4. Discussion

The present study examined whether selective muscle weakness leads to reorganization of the muscle activation patterns underlying target-directed isometric force generation. Using a pharmacological block, brachialis, a major elbow flexor, was weakened in the human arm. Nonnegative matrix factorization was applied to EMG data collected in the pre- and postblock conditions to describe the characteristics of the multimuscle activation patterns in a succinct form (motor modules). We identified five and four motor modules underlying 3D and 2D force target matches under isometric conditions, respectively. The comparison of motor modules identified prior to and after injection of lidocaine showed that the composition of one or two motor modules dominated by activation of shoulder muscles was altered in the postblock condition. For the 2D protocol, we also compared pre- and postblock tuning directions and tuning areas for each muscle. While the spatial tuning direction of individual muscle activation was largely unchanged, the tuning areas of BRA agonist and antagonist muscles typically increased and decreased, respectively, as a compensatory mechanism. The tuning areas of other muscles were altered in a subject-dependent manner. Overall, the present study is the first that provides evidence that selective muscle weakness can induce alternations in muscle coordination in the human arm.

### 4.1. Data Interpretation considering Subject Specificity

The effects of the muscle block in this study exhibited substantial subject specificity. For example, the direction of change in tuning area was not always consistent across subjects. Table 1 shows that no two participants demonstrated the same adaptation of muscle activation in the postblock condition. In addition, in which motor modules were altered after injection of lidocaine was subject-dependent, motor modules with activation of shoulder muscles were most frequently altered postblock. Despite a change in the activation of multiple muscles and motor modules in the postblock condition, the kinetic output (i.e., the magnitude and direction of force targets) met the requirement of the task and was unchanged.

Large intersubject variability in the change in muscle activation patterns and underlying motor modules has also been reported in previous studies which induced pain using saline injection in a single muscle [25, 26]. Muceli et al. [26] found that injection of saline into the anterior head of deltoid resulted in subject-dependent alterations in the composition of motor modules identified during reaching. Similar to the present study, subject-dependent alterations in motor modules were also found for the adjacent joint (i.e., elbow joint).

The mechanisms underlying the response specificity found in our study remain unclear. In an earlier study, we found that the motor modules underlying isometric force generation were generally similar across subjects [39]. A limitation of the current experimental protocol was that the efficacy of the brachialis block varied widely across subjects; the decrease of the tuning area of the injected muscle ranged from 48% to 100%. In turn, this variability would affect the compensatory changes in the activation of other agonistic muscles (elbow flexors) following the lidocaine injection, though the tuning area of the muscles increased in most cases (Table 1). Since some agonists of the injected muscle were biarticular muscles (i.e., BIm and BIlat), the tuning area of shoulder muscles would be affected as a secondary compensatory mechanism, which would involve more variability. In fact, the magnitude of the alteration in the motor modules with shoulder muscle activation was highly variable across subjects (Figures 5(a) and 5(b)).

One could argue that the difference in the composition of motor modules following selective muscle weakness might be attributed to adaptation over the course of the experiment. To partially address this issue, we randomly assigned three of the six repetitions performed for each target in the 2D protocol to each of two datasets. Cross-reconstruction VAF values for the pre- or postblock condition within the same subject were not statistically different (*p* > 0.05). This result indicates that within-subject variability across trial repetitions in either the pre- or postblock condition was negligible, suggesting that the major variability between the pre- and postblock data was related to the block and not motor learning.

### 4.2. Motor Modules and Dimensionality Reduction Techniques

We used a dimensionality reduction technique to quantify intermuscular coordination in pre- and postblock conditions. Our results showed no changes in the dimensionality (i.e., the number of motor modules) following the muscle block, but there were small changes in the composition of motor modules. The fact that the composition of motor modules identified under isometric force target matches was changed after selective muscle weakness can be interpreted in several different ways.

First, the alterations in the composition of motor modules may have implications for neural control of movement via activation of a few motor building blocks (muscle synergy theory). Muscle synergy theory posits that muscle coordination is achieved via hard-wired neural circuits that activate multiple muscles simultaneously [71–73]. Rather than controlling each muscle individually, under synergy theory, the central nervous system activates a small number of motor modules to create movement, as motor modules represent the most basic motor commands. In our experiment, we found that the composition of motor modules or muscle synergies changed following muscle block, which could be interpreted as evidence against muscle synergy theory, given that the alterations occurred over a short time frame. However, it is unclear how the motor point block impacted afferent signals that may contribute to synergy structure. Previous studies suggest that the spinal cord contains networks of interneurons each of which activates selective motoneuron populations to produce a particular muscle synergy [74, 75]. In addition to descending signals, the activation of these spinal interneurons is regulated by proprioceptive signals [76], thus potentially providing a mechanism for rapid changes in hard-wired synergies. Previous studies using animal models, however, showed that the composition of locomotor motor modules was conserved even after complete deafferentation in the frog hindlimb [64]. Whether this is also true for the human arm remains unknown.

Alternatively, previous studies suggest that motor modules obtained from factorization algorithms applied to EMG signals might reflect the task and/or limb biomechanical constraints as well as the characteristics of EMG patterns as an input data of the algorithm [17, 77, 78]. Cadaveric experiments of the hand and simulation studies of the lower extremity have shown that constraints that stem from the task selection and/or limb biomechanics produced synergistic muscle coactivation patterns even though individual muscle control was assumed [77]. Thus, the changes in motor modules in the present study may simply reflect the impact of postblock alterations of limb mechanics on nonsynergic motor control.

Several contrasting interpretations are conceivable as well. For example, altered motor modules may imply that subjects simply chose different muscle activation strategies before and after the block. Different strategies may appear as different motor modules or slightly altered motor modules in the postblock condition.

Another explanation relates to the unsupervised learning technique, nonnegative matrix factorization (NNMF), that we applied to the EMG data to identify motor modules in the study. In particular, NNMF may suffer from a limitation of unsupervised learning, which means that there simply is no correct, validated data to establish the existence of neurally coded motor modules. Thus, NNMF may not be able to correctly identify motor modules, due to intrinsic limitations of matrix factorization, limitations on the number of muscles that can be recorded from [17], or limitations in experimental design and sampling of the feasible muscle activation space. However, if muscle activation had been unchanged following the block, the method, regardless of its limitations, would have indicated that pre- and postblock synergies were unchanged, which is different from the results in the present study. So this explanation would be less supported by the presented data.

We reason that the alteration of motor modules following selective motor point block could be attributed to both neural and task/biomechanical constraints as well as the characteristics of the factorization algorithms we applied to the data, to a different extent, respectively. The association between the activation of neuroanatomical constraints with characteristic intermuscular coordination patterns has been widely tested in the animal model [68, 75, 79]. The perspective that the precise alternations in motor modules may be due to the subject-specific difference in the limb biomechanics is congruent with the previous findings [70, 77]. In relation to the debate on the origin of muscle synergies, the current study design is not optimal to test or nullify the muscle synergy theory. Rather, this study is the first that used the factorization algorithms to quantify the potential changes in muscle coordination including both superficial and deep muscles following selective motor point block.

### 4.3. Relevance to Orthopaedic and Neurological Issues

Common everyday activities involve reaching toward an object of interest by coordinating the mechanically redundant muscles of the limb to generate appropriate forces. Orthopaedic injuries and procedures, including tendon rupture and tenotomy as well as neurological disorders, such as stroke, motor mononeuropathy, plexopathy, and radiculopathy, often induce muscle weakness. For example, approximately 77% of stroke survivors have muscle weakness in the upper extremity, which is a critical limiting factor of their activities of daily living [80].

The results of the present study show that muscle coordination in a mechanically redundant muscle set is altered by selective muscle weakness induced in the human upper extremity. The compositions of motor modules in the presence of muscle weakness were altered, reflecting a redistribution of muscle contributions to the end-point force. Our results suggest that the aforementioned procedures may lead to alterations in the coordination of muscles at multiple joints. To date, this study is the first to show how muscle coordination of both superficial and deep muscles of the human arm is altered in the presence of selective muscle weakness, one of the consequences of orthopaedic and neurological impairment.

### 4.4. Future Directions of Research

While muscle redundancy has been examined in the context of motor modules [35, 63, 68, 81–85], relatively little attention has been given to the robustness of limb force production to dysfunction of a single muscle [86]. Cadaveric and simulation studies of human fingers and the lower limb suggest that muscle redundancy provides surprisingly little robustness to muscle loss [86]. However, to our knowledge, the robustness of force generation in the human arm has not been evaluated *in vivo*, which can be addressed as a future study.

The extent to which the feasible force range (FFR) and active range of motion (AROM) is reduced by induced or naturally occurring muscle dysfunction is likely to be dependent on the underlying neuromuscular control strategy [77]. Specifically, motor module-based muscle control (synergic control) is likely to result in more severely reduced FFR and AROM following muscle loss, relative to alternative strategies such as individual muscle control (nonsynergic control). Examinations of the relationships among muscle redundancy, neuromuscular control strategies, and robustness of force and motion generation to dysfunction of a single muscle have critical clinical implications for orthopaedic procedures and musculoskeletal injuries that result in selective muscle weakness or loss. These injuries or procedures include muscle flaps for body reconstruction [87, 88] and tendon transfer [89, 90], in addition to tendon rupture [91–93] and tenotomy [94–96].

Clinically, the detailed knowledge of the degree to which the FFR is still achieved after inducing muscle weakness has critical implications, especially for orthopaedic procedures such as tenotomy. For example, debridement and arthroscopic release of the long head of the biceps tendon (LHBT) from the labrum has been a current trend to treat older adults with shoulder pain and morbidity related to the shoulder [97]. The required dissection of the LHBT, or tenotomy, can decrease elbow flexion strength [95], as well as decrease supination strength [91]. However, the effects on combined load conditions, that is, the FFS, have not been evaluated. The scientific findings on muscle-specific robustness of force generation to focal muscle paresis, as outputs of future studies, can serve as a foundation to make evidence-based clinical decisions that minimize functional loss.

This study provides a novel approach to quantitatively assess available compensatory strategies for selective muscle weakness, led by experimentally identified neuromuscular constraints, during isometric force generation but not active arm reaching. We conjecture that our study can be extended to a future direction by integrating experimental finding, an advanced analytic tool to identify neuromuscular coordination strategies, and complementary musculoskeletal simulations that are constrained by the experimentally identified motor modules. The musculoskeletal simulations will also use novel methods for modeling a neuromuscular control and provide optimal methods for predicting muscle recruitment with and without impairment. In this study, this method was not used because this is out of the major scope of the current work.

## 5. Conclusion

Neurological sequelae may result in both muscle weakness and impaired muscle coordination. The relationship between the deficits can be observed but remains unclear. The present study is the first that shows that selective muscle weakness induces reorganization of muscle coordination that includes activation of both superficial and deep muscles in the human arm, by using ultrasound-guided, pharmacological muscle block, and dimensionality reduction tool. Though the alteration in the muscle coordination included subject-dependent variability, most of the participants showed the change in the tuning areas of the injected and other muscles as well as the modification in the composition of motor modules without recognizable changes in the task demands in the postblock condition. The results suggest that selective muscle weakness induces the modulation of individual muscle weights that can lead to reorganization of muscle activation as a compensatory mechanism to accomplish the task requirement.

## Figures and Tables

**Table 1:** Changes in preferred direction and tuning area.

	Muscles												
Subjects	BRD	Blm	TRIlong	TRIlat	AD	MD	PD	PECTClav	BIlat	ANC	PRO	SUP	BRA
Preferred direction (Deg)													
M1	0	−6^∗^	−5^∗^	−2	−13	−38^∗^	−7^∗^	8	−9^∗^	−7	−13	5^∗^	−12
M2	−6	17^∗^	−8^∗^	−2	−4	0	−1	2	0	3	2	5	4
M3	−3	−1	−1	−4	2	13	1	−2	−10	4	−18	−5^∗^	−8
M4	−4	−12	7	1	−8	12	4	2	−9	−11	−10	−7	−5
M5	−2	3	−8^∗^	−8	5	−35^∗^	−4	6	3	−2	0	—	6
M6	−2	−2	2	−1	4	11	−1	0	0	−1	1	0	−8
M7	−3	−3	1	1	2	1	1	—	−2	2	−2	—	−16
Tuning area (%)													
M1	35^∗^	158^∗^	−32^∗^	−11	431^∗^	27	11	76^∗^	82^∗^	−22^∗^	42^∗^	−9	−81^∗^
M2	106^∗^	−12	−26^∗^	−43^∗^	7	−21	−24^∗^	−8	42^∗^	−12^∗^	9	−83	—
M3	25^∗^	106^∗^	6	72^∗^	−33^∗^	111^∗^	108^∗^	−17	71^∗^	−25^∗^	−58	18	−99^∗^
M5	62	28	59	11	−14	−19	−30	180	96^∗^	49^∗^	25	—	−1000
M6	76^∗^	107^∗^	11	−9	19	92	53^∗^	21^∗^	98^∗^	−15	38	−44	−93
M7	36^∗^	57^∗^	2	−41^∗^	−54^∗^	23	−8	—	5	77^∗^	490^∗^	—	−48

^∗^ *p* < 0.05. The negative sign of the change in preferred direction indicates clockwise rotation of the direction following injection of lidocaine. Em dash (—) indicates missing measurement.

## Data Availability

The data will be available based on personal request for potential productive collaborative opportunities.
